# Laboratory data of dog rabies in southern Cameroon from 2010 to 2013

**DOI:** 10.1186/1756-0500-7-905

**Published:** 2014-12-12

**Authors:** Serge Alain Sadeuh-Mba, Laura Besong, Maurice Demanou, Sévérin Loul, Amadou Nchare, Richard Njouom

**Affiliations:** Service de Virologie, Centre Pasteur du Cameroun, Rue Dunant, B.P. 1274 Yaounde, Cameroon; Ministry of Livestock, Fisheries and Animal Industries, Yaounde, Cameroon

**Keywords:** Rabies, Dogs, Cameroon, Surveillance

## Abstract

**Background:**

Dog rabies is endemic in most African countries and the risk of human rabies is estimated to be high in Cameroon according to WHO estimations in 2010. This study aimed to describe the circulation rabies virus (RABV) among dogs in the southern regions of Cameroon from 2010 to 2013 in a context, where mass vaccination campaigns are launched annually in order to control rabies in domestic animals including dogs and cats.

**Findings:**

From 2010 to 2013, 93 animal specimens (dogs: 91, monkey: 1, pig: 1) originating from the southern regions of Cameroon were collected and tested for rabies virus at the Centre Pasteur of Cameroon by fluorescent antibody test (FAT) and virus isolation. Of the total dog specimens, 69.2% (63/91) originated from the central part of the southern regions and 50.5% (46/91) were from the capital city Yaounde. Overall, 74.2% (66/89) of dogs’ specimens that could be tested were found rabies-positive while specimens from the monkey and pig were tested negative. Overall, dog rabies was repeatedly detected in the southern regions of Cameroon especially in the nation capital, Yaounde even though low specimen submission and geographic bias did not permit major conclusions about its actual rate, geographical and over time distribution.

**Conclusions:**

The results of this study indicate that rabies is endemic in the dog population which is of public health concern. Therefore, coordinated rabies control program should be conducted to reduce the rabies incidence in dogs and in humans. In addition, proper rabies surveillance program including reporting system should be established to monitor the success of the control program in Cameroon.

## Findings

Rabies is a viral zoonotic infection commonly caused by the rabies virus (RABV), but also by other negative strand RNA viruses belonging to the *Lyssavirus* genus in the *Rhabdoviridae* family [1, 2]. RABV is transmitted to healthy mammals through the exposure to the saliva of infected mammals of the same or a different species mainly through bite or scratch. Rabies surveillance and control programs based on effective vaccination and dog population management have led to the elimination of rabies in domestic animals in Western Europe, North America as well as in some countries in Asia. However, rabies remains a public health issue in developing countries where dogs remain the main reservoir and vector of disease transmission to humans [3–5]. It is estimated that about 55,000 human deaths annually are caused by rabies infection worldwide and 44% occur in Africa [3, 5].

**Figure 1:**
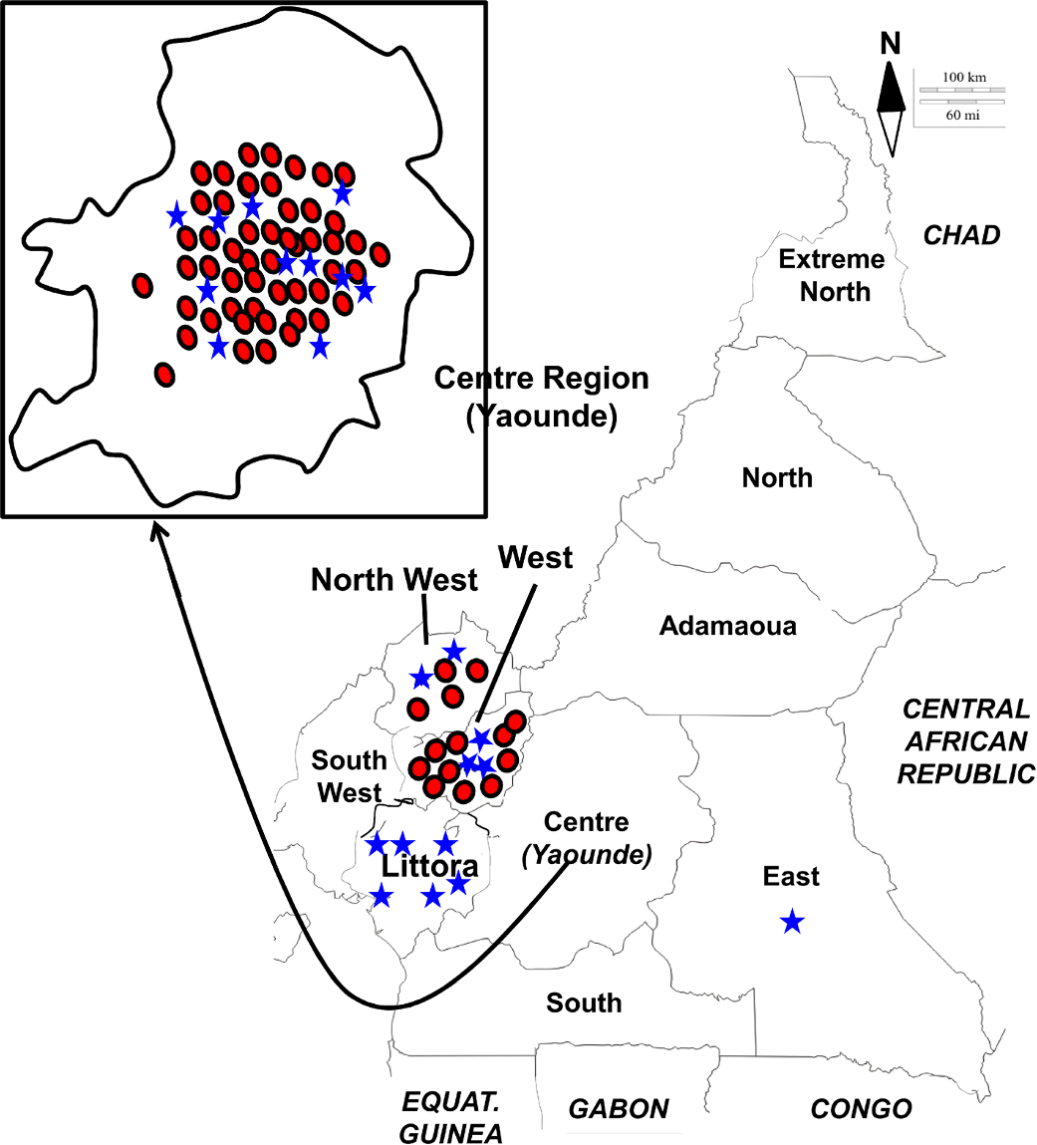
**Distribution of the dog specimens tested positive (red-filed circles) and negative (blue stars) for rabies, in Cameroon, 2010–2013.**

There is very limited data about the frequency of both animal and human rabies in Cameroon. The unique report documenting the situation of human and dogs rabies in Cameroon, from 1990 to 1999, indicated that dogs were the primary source of human rabies in Cameroon [6]. This retrospective review reported that a total of 721 quarantined suspected dogs died during observation among which 330 (45.8%) were laboratory-confirmed positive for rabies. The endemicity of canine rabies in Cameroon led to the adoption of the 2001 law defining the general strategy for the control of animal contagious diseases, including rabies. Since then, animal health professionals are expected to implement compulsory notification of animal rabies cases and owners have to get their dogs and cats vaccinated against rabies. Since 2009, a nationwide mass vaccination campaign of dogs and cats against rabies was launched on a yearly basis during the commemorations of the international rabies day. These are carried out by organizing fixed vaccination posts and by providing rabies vaccine and administration cost at reduced prices for dog owners (2 rather than 10$). However, the efficiency of rabies control interventions was not monitored scientifically. Although the animal and human public health authorities are aware of the need for the control of dog rabies, the active rabies surveillance system is not yet in place. In Cameroon, any suspected rabies specimen originating from the 7 southern regions of Cameroon are tested at the Centre Pasteur du Cameroon (CPC) in Yaoundé, while the specimens originating from the northern regions of Cameroon (Extreme North, North and Adamaoua) are referred to the national veterinary laboratory (LANAVET) (Figure 1). This study aimed to describe the situation of animal rabies in southern Cameroon through the retrospective analysis of data from CPC’s routine specimens tested during 2010–2013.

### Specimens and methods

Animal specimens were collected with the approval of the Cameroonian Ministry of Livestock Fisheries and Animal Industries within the framework of the rabies diagnosis in Cameroon. Specimens analyzed were obtained by convenience sampling since there is no system in place to actively collect animal specimens from the veterinary networks for rabies diagnosis in Cameroon. Thus, passive surveillance during 2010–2013 was maintained by veterinarians and animal health officers who sent specimens from animals that expressed rabies symptoms, appeared sick and/or had bitten someone, for rabies testing at CPC. The entire head of the suspected animals were sent frozen to CPC where the brain was extracted, kept at +4°C and tested within 48 h.

Detection of rabies nucleocapsid was performed on the brain postmortem biopsy by fluorescent antibody test (FAT) using rabbit IgG against RABV nucleocapsid (Bio-Rad, Marnes-la Coquette, France) as previously described [7]. The negative FAT results were further confirmed by virus isolation which was performed by inoculation into Murina neuroblastoma cell cultures as described elsewhere [7].

### Geographic origin of the specimens

From 2010–2013, 93 specimens (dogs: 91; monkey: 1 and cow: 1) were submitted to CPC for rabies testing. Of the 91 dog specimens submitted, from the southern regions of Cameroon including Center, East, Littoral, North West and West, two samples were not suitable for laboratory testing (Table 1). The samples submitted for testing from the East, Littoral and North West regions were 1, 6 and 6, respectively while no specimens were received from South and South West regions. Overall, 70.8% (63/89) of the dog specimens analyzed originated from the Centre region among which 73.0% (46/63) were sampled in the nation capital Yaounde where the CPC is located (Figure 1). Two dog specimens that originated from the Center and West regions could not be tested due to poor condition. The existing veterinary network in Cameroon was found to be efficient for conservation and shipment of specimens for laboratory testing.

**Table 1:** **Rabies laboratory diagnosis in domestic animals, Cameroon, 2010–2013**

Species	Specimens		Tested positive (%)
	**Received**	Tested*	
dogs			
2010	29	29	19 (65.5)
2011	14	14	10 (71.4)
2012	23	22	20 (90.9)
2013	25	24	17 (70.8)
**Total dogs**	**91**	**89**	**66 (74.2)**
monkeys			
2010	1	1	0
cows			
2010	1	1	0
**Total (all)**	**93**	**91**	**66 (72.5)**

*Two dogs samples received in 2012 and 2013 from the Center and West regions were not tested because of poor condition.

Totals are highlighted in bold.

### Geographic and overtime distribution of positive samples

**Figure 2:**
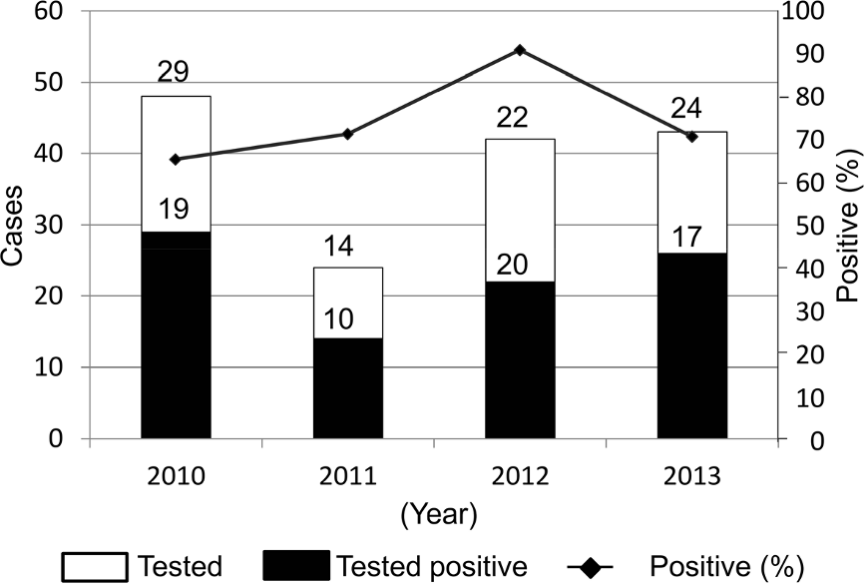
**Rabies laboratory diagnostic in dogs, southern Cameroon, 2010–2013.**

Overall, 74.2% (66/89) of brain specimens from dogs were found rabies-positive using FAT while specimens from the monkey and pig were tested negative (Table 1). Moreover, virus isolation was negative for all 25 specimens whose FAT was initially negative. The percentage of positive specimens varied across the 4-years period from 65.5 % (19/29) in 2010 to 90.9% (20/22) in 2012 (Figure 2). The numbers of specimens submitted for diagnosis were very low to estimate the actual rate and the seasonal pattern of dog rabies. However, dog rabies was laboratory confirmed in 3 regions of the southern regions of Cameroon including Centre, West and North West (Figure 1). Concerning the capital city Yaoundé in particular, 78.2% (36/46) of specimens were tested positive for RABV antigen: 7, 3, 15, and 11 specimens in 2010, 2011, 2012 and 2013 respectively. These data provide evidence of the risk of rabies acquisition from dog bites in at least some areas of Cameroon including the nation capital despite increasing awareness and efforts towards rabies control.

The percentage of rabies-infected dogs over the 2010–2013 period (74.2%; 66/89) was apparently higher than the one reported during the 1990–1999 period (45.8%; 330/721) [6]. The higher frequency in this study may be due to the fact that most specimens were sampled in Yaounde and where thus sent to the laboratory in good conditions. This difference may also be due to more accurate clinical diagnosis by veterinarians/and technicians in the field. As previously reported in Cameroon [6], dog rabies seemed to be more frequent in the urban areas than rural areas. However, this observation could be a result of a bias caused by the poor accessibility to the diagnostic laboratory at CPC and also the lack of knowledge of the rural population about dog rabies. There is a need of increased sensitization of the population, especially in rural areas, about the rabies risk. Another factor that may have account for underestimation of dog rabies in rural areas is the affordability of sample shipment from periphery levels to the CPC laboratory at the central level. Furthermore, the fact that dog owners have to pay for the treatment of bitten individuals constitutes a factor of underreporting to the local health authorities. As reported in other African countries [5, 8], reporting bias, lack of sample submission from distant regions especially rural areas and the lack of awareness campaigns may have resulted to the underestimation of the overall rate of dog rabies in the southern Cameroon.

Despite the main intervention of animal health authorities through mass vaccination campaigns of dogs and cats against rabies launched regularly on the international rabies day, dog rabies remains a threat to human health in Cameroon. Theses campaigns have not been as effective as expected from previous reports [9, 10]. Till now, interventions for the prevention of human rabies through dog rabies control have been based on very limited data generated from passive surveillance. To be more effective future vaccination campaigns against dog rabies need to be accompanied by a scientific evaluation in the light of data generated from an active rabies surveillance system and further field studies. In particular, these later should address the specific gaps in the knowledge about the awareness of the human populations, dog demography and ecology as well as the optimal timing and periodicity of vaccination campaigns.

### Ethical approval

Not required. Animal specimens were collected with the approval of the Cameroonian Ministry of Livestock, Fisheries and Animal Industries within the frame of the rabies surveillance in Cameroon.
